# False Impressions

**Published:** 1888-12-22

**Authors:** Caroline Fothergill

**Affiliations:** Author of "An Enthusiast," "A Voice in the Wilderness," etc.


### False Impressions.

By Caroline Fothergill, Author of "An Enthusiast," "A Voice in the Wilderness," etc.

*(Continued from p. 174.)*

### Chapter XII.—A Dog's Death.

George drew near the house and looked at it. It was an ordinary working man's dwelling of, perhaps, rather the better class : two rooms up and two down, with a back kitchen on the ground floor. Glancing in at the front window, he saw that the room looked bare and empty. Probably the greater part of the furniture had been sold or pawned to meet the more pressing daily wants as they arose. This man and his wife had no children, so they had been able to cling to their old home longer than if they had had the additional expense of a family. No doubt, as they could count Miss Stanford among their friends, they would be able to tide over the bad time.

George looked at it for a moment, and then retired down the street, and leaned over the canal wall, looking down into the water and wondering how long she would be. He had gone first out of Dacre Street, but the canal passed along the end of it, and he would see her leave the house and be able to follow her again.

He sat for some time thinking about her. She had proved herself strong, true, and courageous in spirit. He did her lowly homage, and most bitterly did he repent his former attitude towards her, which had been kept up so long that the chances of his ever being able to alter it for one more in keeping with his feelings were very small indeed. He was eager for a change, but he thought, angrily, she was not. He had carried things too far, and had estranged her beyond any hope of reconciliation. And in this wretched conventional nineteenth century, he told himself, desperately, there would never come an opening for him to show her how he felt. If he could only do something, perform some service for her, and so convince her of the reverence in which he held her! But that was impossible, he need hope for no such favour from fate.

All this time he stayed where he was, thinking of Beatrice so intently that he forgot to turn his face to Dacre Street, that he might see her when she left the house where she was. The street was quiet, and his meditations were not disturbed until a sudden uproar roused him, and he looked up.

A number of boys were coming along the canal bank below him. By drawing back a little he could see without being seen, and he watched them with some curiosity. They were shouting and gesticulating, after the manner of boys, and mingled with their shouts was another sound which changed George's curiosity into suspicion, and made him look at them very sharply indeed. In their arms (for it would have been difficult to say who or how many were carrying it) was a wretched dog, almost a puppy, which was crying loudly with fear or pain. Looking closer, he felt inclined, from the attitude of the dog and some suspicious-looking stains on the clothing of one of the boys, to think the animal was in pain, and he was just wondering what they were going to do with it, and whether it was worth while following them - that meant giving up all hope of going with Beatrice, and he hesitated about making the sacrifice—when two more boys came into view, carrying a ferret in a cage. The moment he saw them, and saw they were going in the same direction as the other lads, his hesitation vanished, his scruples were overcome, and giving utterance to something which sounded very much like an oath, he was just going to drop over the wall on to the canal bank when a third party appeared, and this time he uttered a low whistle and slipped quickly back out of sight. The new-comer was none other than Miss Stanford, looking very pale and determined, and walking with a swift, even step which somewhat tried the small, tearful boy who trotted at her side. The child was speaking between his sobs, and George also caught the few questions which Miss Stanford asked. From these and the conclusions he drew from what he had seen and heard before, he was soon tolerably clear that the dog belonged to this child, that it had been taken from him by older and stronger boys, who had given it over to the ferret, thereby testifying to the noble love of sport inherent in every true-born Briton. They had probably been disturbed in their amusement, but having secured both their victim and its adversary were off to finish the scene in some place where they would be secure from interruption. How Beatrice came to be mixed up with it he neither knew, nor sought to know, probably—and in his supposition he was right—she had concluded her visit, left the house, encountered this crying child, inquired the cause of his tears, and hearing it, had hurried off in her impulsive way to avenge him.

As soon as George saw Beatrice, he determined not to interfere himself, but to see to what purpose she would interfere. He therefore dropped over the wall and followed her and her guide at a safe distance.

They did not go far before they came to an archway over the canal, and just on the other side of the archway the boys were gathered in an open space of ground, making noisy arrangements for the renewal of their sport. George halted in the deep shadow of the archway. Here he felt pretty safe from observation, and could himself see everything that went on. He was angry to see that the dog was more hurt than he had believed : one of its legs was broken, and its neck was torn and bloody. Two or three men of a thoroughly low type had joined the group, and were now making bets upon the fight. The boys, flattered by this recognition of their sport from grown-up men, yet apprehensive at the hints which were being thrown out as to the desirability of taking the whole matter out of their hands and conducting it on more scientific principles, were whispering together in a state of complete indecision.

Into this group Beatrice made her way, and George came a step nearer. She stood side-ways, so that he could see her profile; she was very pale, but her mouth was set in a curve of iron determination, and her eyes burned with a sombre fire he had never seen or imagined in them before; her brows were drawn together in a frown. Her sudden appearance created a little stir among the boys and men, one or two edged away as if a sense of shame were beginning to awaken in them.

"Boys," she began, and her voice rang out full and clear and imperious. George smiled as he heard it; that tone was well known to him; it was the first in which he had ever heard her speak. "Boys," she repeated, as if to make sure of gaining their attention, "what are you going to do with that dog?"

There was no definite answer, only a murmur in which the word "ferret" was distinguishable.

"Are you going to give it to that ferret, to be torn to pieces?" she went on, and this time was answered by a tolerably unanimous

"Ay!"

"You will do nothing of the kind," she said. "Give the dog to me."

She stretched out her hand to receive it as she spoke, but, perhaps in obedience to an unseen sign, it was withheld, and one of the men spoke in a coarse, bantering tone, which made George clench his fist, and feel the blood throbbing in his temples.

"Come, missis, dunnot be so hard on th' childer; It's nobbut a bit of spoort."

She turned quickly to them, and George saw that she was trembling with indignation : he hoped she would not lose her self-control.

"As for you," she said, "you are cowards, all of you. These children scarcely know any better, they are only doing what they have seen you do again and again. But you are cowards to stand by and see them do it. You ought to be ashamed of yourselves; if you were men you would be; you would stop it at once."

"Bravo!" said George, beneath his breath. She had spoken strongly, but concisely; she was not losing her self-control, and he wondered what answer she would get.

She got none. Perhaps they were too much astonished at being thus addressed by a woman to speak, although the Mayfield operative is not usually at a loss for words. Beatrice bestowed no more speech upon them, but turned again to the boys and advanced towards the one who held the dog.

"Give me that dog," she said again, and although she did not raise her voice, her tone and manner seemed to compel obedience, for the poor little mongrel was put into her arms silently, without a word from anyone. George, watching the scene with breathless interest, wondered what she would do now. As far as he could see, there was nothing for it but to kill the little creature at once.

Beatrice took it gently, although it shrieked with pain during the transit, and George saw that her eyebrows contracted as if with pain. But she looked the dog carefully over, and did not flinch from the terrible sight it presented.

She held her audience. No one moved or spoke, and when she had finished her inspection, she looked round upon them for a moment before she spoke.

"Do you know what you have done?" she said at last. "When you got hold of this dog it was whole and sound; it enjoyed its life, it had a kind master who was fond of it, and whom it loved, and it was very happy. Now you have so ill-treated and tortured it that the kindest thing is to kill it at once and put it out of its misery."

Her voice was full and deep with indignation, and towards the end it shook a little. The unhappy owner of the poor little beast sobbed bitterly, saying: "Oh missis, missis!" and the dog itself shivered and yelped as if it understood that its fate was being sealed. Otherwise there was complete silence, until one lad, with a sharp, cunning face, said,

"How are you going to kill it, missis? Will you gi' it to t'ferret? t'ferret 'll kill it in a minute."

She turned a look upon the boy, which made him colour and withdraw among the crowd, muttering,

"You know naught about dogs."

While one of the men growled out—

"Shut your mouth, lad!"

Beatrice took no notice of this little bye-play, but said briefly—

"I shall drown it."

No one spoke, and she went on—

"Is there a large stone, or any other heavy thing, at hand?"

There was nothing, and no one offered to bring anything, partly, no doubt, out of natural unwillingness, partly because her proceedings had aroused interest, and no one wanted, by even momentary absence from the scene of action, to run the risk of missing the end of the play. George was just as anxious as the others to see what she would do.

They had not to wait long. Beatrice Stanford was as prompt in action as in thought and speech. She cradled the dog tenderly in one arm, then knelt down in her dress of muslin and lace in the dust and dirt of the canal bank, and then baring one arm—George suddenly saw with a thrill what beautiful arms she had—she set her lips, clenched her other hand, and plunging the dog under water, held it until its struggles had ceased, and she knew it was dead.

As soon as the dog's body had for good and all sunk beneath the water the scene changed almost instantaneously. The crowd that had watched this summary administration of justice and mercy cleared away as if by magic. Not one stayed behind, and Beatrice was left alone to rise to her feet and stagger to the wall against which she leaned for support.

She was very much upset, now that the strain was removed, the reaction came, and she sobbed almost hysterically. She so evidently believed herself to be alone, that George hesitated as to whether he should go to her or not. But when he saw her by a tremendous effort check her tears, dry her eyes, and with tremulous and halting steps begin to walk back to Dacre Street, he could remain hidden no longer, but stepped forward and placed himself at her side.

"Lean on me Miss Stanford," he said quietly, "you cannot walk alone."

She turned and looked at him without speaking; then the hot colour flushed her face, driving away the pallor, which had begun to alarm him.

"Mr. Murray!" she exclaimed, "How did you get here?"

"I followed you," he said, meeting her eyes directly, "you told me not to come, but I disobeyed you; I am glad now that I did."

"Yes," was her unexpected reply as she let him raise her hand and place it on his arm, "I am very glad you did."

And, indeed, she was more thankful than she could say for his sudden and unexpected appearance in this dreary wilderness, and she was not sufficiently mistress of herself to conceal her relief and satisfaction. George's heart beat high at her admission.

"Sit down for a minute," he said; "the wall is low here, and you cannot walk."

"Oh, yes, I can," she answered, with a faint smile. "I am only a little upset. It was very trying to have to destroy the dog myself; I never killed anything in my life before; but I believe it was necessary."

"I saw it was necessary, but I did not know if you would have the nerve. It was a very brave thing to do. You are the bravest woman I know. I doubted you once, and I beg your pardon for it humbly."

The tone in which he spoke brought Beatrice to herself again. She suddenly realised how she had yielded to him, and she withdrew her hand from his arm, saying—

"I can walk alone now; I need trouble you no further."

He saw the change, and bit his lip. It was odd how this reserved, reticent man was constantly letting his tongue run away with him when he was talking to Beatrice. After this they walked almost in silence to the nearest cab stand. He put her into a cab, and stood on the pavement watching her drive away.

*(To be continued.)*

